# Spurious Hyperphosphatemia From Alteplase‐Treated Catheter: A Case Report and Literature Review

**DOI:** 10.1002/ccr3.70665

**Published:** 2025-07-27

**Authors:** Ahmad Matarneh, Sundus Sardar, Omar Salameh, Muhammad Abdulbasit, Wardah Iqbal, Monika Koirala, Taryn Millete, Ronald Miller, Nasrollah Ghahramani, Navin Verma

**Affiliations:** ^1^ Department of Nephrology Pennsylvania State Milton S. Hershey Medical Center Hershey Pennsylvania USA; ^2^ University Health Truman Medical Center Kansas City Missouri USA; ^3^ Akthar Saeed Medical and Dental College Lahore Pakistan; ^4^ Internal Medicine Residency Program UPMC‐Lititz Lititz Pennsylvania USA; ^5^ Pennsylvania State Milton S. Hershey Medical Center Hershey Pennsylvania USA

**Keywords:** electrolyte imbalance, end‐stage renal disease, hyperphosphatemia, renal dialysis, tissue plasminogen activator, vascular access devices

## Abstract

Falsely elevated phosphate levels can occur when blood is drawn after alteplase use in dialysis catheters due to phosphorus‐containing excipients. Recognizing spurious hyperphosphatemia is important to prevent misdiagnosis, avoid inappropriate treatment changes, and ensure accurate phosphate monitoring in patients undergoing maintenance hemodialysis.

## 1 Introduction

Phosphorus balance is a concern in chronic kidney disease (CKD) and end‐stage renal disease (ESRD) patients, as hyperphosphatemia is strongly associated with vascular calcification, secondary hyperparathyroidism, and increased cardiovascular risk [1]. In hemodialysis patients, phosphate levels are typically controlled through dietary restrictions, phosphate binders, and adequate dialysis [2]. However, laboratory errors may sometimes present falsely elevated phosphate readings, leading to unwarranted treatment adjustments or heightened clinical concern [3].

Among the various causes of spurious hyperphosphatemia, contamination from catheter‐based thrombolytic agents is an underrecognized but clinically significant factor. Alteplase, a recombinant tissue plasminogen activator (tPA), is routinely used to clear occluded central venous catheters (CVCs) and tunneled dialysis catheters [4]. It is supplied as a lyophilized powder containing arginine, phosphoric acid, and polysorbate 80, and is reconstituted with sterile water. These phosphate‐containing excipients help stabilize the drug. If not fully flushed prior to blood sampling, they may contaminate the sample and lead to falsely elevated phosphate measurements. If a blood sample is drawn before the complete flushing of alteplase and its excipients from the catheter lumen, laboratory abnormalities may arise, mimicking true electrolyte derangements [5]. This case report illustrates how blood contamination from residual alteplase led to an erroneous phosphate elevation in a hemodialysis‐dependent ESRD patient with otherwise well‐controlled phosphate levels.

## 2 Case History and Examination

A 70‐year‐old male with a history of ESRD on maintenance hemodialysis three times per week presented with an unexpected elevation in serum phosphate levels. His medical history was significant for type 2 diabetes mellitus (T2DM), hypertension, dyslipidemia, gout, and prior liver transplantation. He had been on paracalcitol 4 μg as part of his dialysis regimen for the management of secondary hyperparathyroidism.

His monthly laboratory assessments consistently showed well‐controlled phosphorus levels without the need for phosphate binders, maintained through dietary measures alone. Over the past few weeks, he had experienced recurrent dialysis catheter dysfunction, necessitating multiple alteplase (2 mL per port tPA) instillations to restore catheter patency.

## 3 Differential Diagnosis, Investigations, and Treatment

During a routine hemodialysis session, blood samples were drawn from his tPA‐treated catheter, revealing a serum phosphorus level of 9.4 mg/dL (reference range: 2.5–4.5 mg/dL). Given his stable phosphorus control in prior assessments and the absence of symptoms of hyperphosphatemia (e.g., pruritus, muscle cramps, or soft tissue calcifications) as well as strict adherence to dietary restrictions, this result was suspected to be an outlier. A repeat phosphate measurement from a peripheral venous sample returned to a normal level of 3.5 mg/dL, confirming spurious hyperphosphatemia likely due to residual alteplase contamination in the catheter (Table 1). Same day Laboratory Comparison Between Temporary Dialysis Catheter (TDC) and Peripheral Samples.

**TABLE 1:** Same‐day laboratory comparison between temporary dialysis catheter (TDC) and peripheral samples.

Lab test	Reference range	Peripheral	tPA treated TDC
Na	136–145 mmol/L	140	135
K	3.5–5.1 mmol/L	4.1	4.0
Cl^−^	98–107 mmol/L	101	99
HCO_3_	22–29 mmol/L	27	24
BUN	6–23 mg/dL	26	29
Cret	0.70–1.30 mg/dL	7.03	7.40
Ca	8.4–10.2 mg/dL	8.9	8.7
PO_4_	3.5–5.3 mg/dL	3.1	9.4 (Critical high)

**FIGURE 1:**
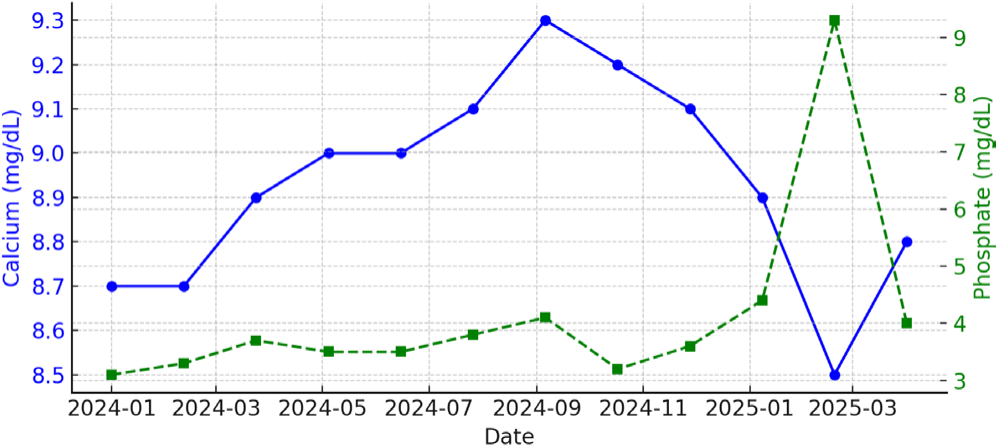
Calcium and phosphorus trends over time.

**FIGURE 2:**
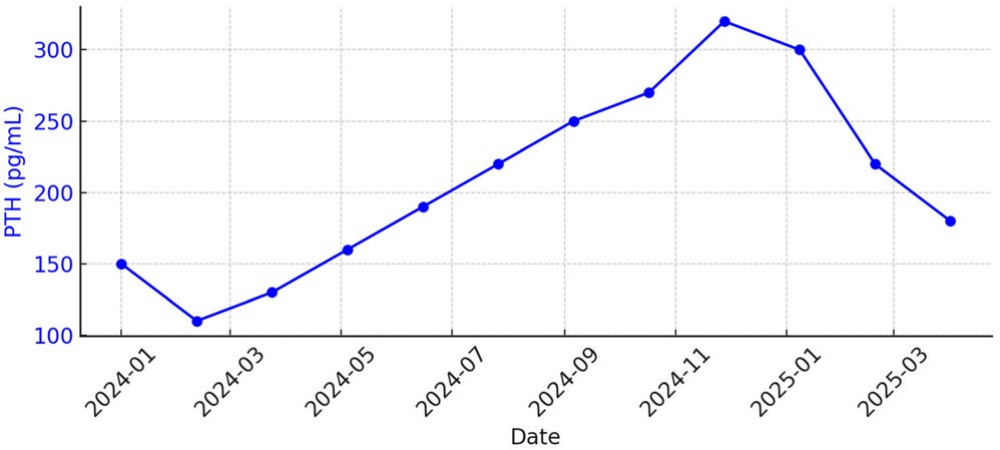
Parathyroid hormone trends over time.

**FIGURE 3:**
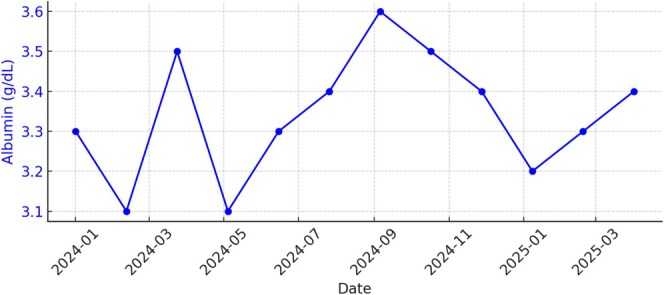
Albumin trends over time.

Additional laboratory results during this period are shown in the graphs below (Figures 1, 2, 3):

- Calcium: Stable between 8.8 and 9.2 mg/dL
- Parathyroid Hormone (PTH Intact): Progressively rising from 120 to over 320 pg/mL, indicating worsening secondary hyperparathyroidism (sHPT)
- Albumin: Stable at 3.8–4.0 g/dL, reflecting good nutritional status

## 4 Outcome and Follow Up

Despite the gradual increase in PTH over time, his calcium and phosphate remained largely controlled, and he continued on paracalcitol therapy without requiring additional interventions at that time.

Given the recurrent catheter dysfunction and frequent tPA instillations, the nephrology team emphasized the importance of proper catheter flushing before blood draws to prevent contamination‐related laboratory artifacts. Consideration was also given to alternative vascular access options, given his ongoing catheter‐related issues.

## 5 Discussion

Hyperphosphatemia in ESRD is typically due to impaired renal phosphate excretion, necessitating management through dialysis, dietary restriction, and phosphate binders [6]. However, falsely elevated phosphate readings can occur due to pre‐analytical variables, including hemolysis, sample contamination, and interference from anticoagulants or thrombolytic agents [7]. In this case, residual alteplase within the catheter lumen likely contaminated the blood sample, leading to an artificially elevated phosphate measurement.

Alteplase (Cathflo) is a tPA formulated with stabilizing additives, which may include phosphate‐based buffers [8]. When blood is drawn from a catheter without complete flushing of residual alteplase, these additives can mix with the sample, resulting in a laboratory artifact that mimics true hyperphosphatemia [4]. In ESRD patients, where phosphate control is a cornerstone of dialysis management, such falsely elevated values can mislead clinicians, prompting unnecessary treatment modifications or additional testing. Alteplase can cause side effects such as bleeding, allergic reactions, hypotension, and rarely, anaphylaxis or arrhythmias. Recognition of spurious hyperphosphatemia is essential to prevent misdiagnosis, patient anxiety, and inappropriate interventions [9]. In dialysis‐dependent patients, an isolated elevation of phosphate in the absence of clinical symptoms or corroborating biochemical abnormalities should raise suspicion for analytical interference.

We conducted a brief literature review (Table 2) on spurious hyperphosphatemia and identified several case reports. These reports emphasize the clinical relevance of recognizing catheter‐related lab artifacts in ESRD patients, especially those undergoing dialysis through central venous catheters. Most highlight isolated phosphate elevations without other metabolic derangements, shedding light on the importance of correlating lab results with clinical context, highlighting its occurrence in patients with central venous catheters, particularly those maintained with anticoagulants such as heparin or alteplase. The primary mechanisms leading to falsely elevated phosphate levels include the following:

1. Contamination of blood samples—Residual phosphate‐containing solutions, such as heparinized saline or alteplase (which has high phosphate content in its excipient), can mix with blood samples if catheters are not adequately flushed before drawing.
2. Phosphate‐containing anticoagulants—Heparin and citrate solutions used for catheter locks or dialysis circuits may introduce excess phosphate into collected blood samples.
3. Pre‐analytical errors—Inadequate flushing of central venous catheters before blood sampling increases the likelihood of spurious hyperphosphatemia, leading to potential misinterpretation of laboratory results.

**TABLE 2:** Summary of reported cases of spurious hyperphosphatemia due to catheter‐related contamination.

Publication	Case presentation	Findings
A [9]	Case report of a patient with an indwelling catheter maintained with heparinized saline. Investigated cause of spurious hyperphosphatemia by analyzing blood samples drawn from the catheter and assessing contamination.	Minimal contamination of blood samples with heparinized saline used in catheter maintenance can cause significant increases in measured phosphate levels, leading to pseudohyperphosphatemia. Emphasized strict blood drawing procedures.
B [10]	Case report of a pediatric patient with an alteplase‐locked central venous catheter. Investigated cause of spurious hyperphosphatemia by analyzing blood samples and phosphorus content in alteplase.	Contamination of blood samples with alteplase, which contains high phosphorus in its excipient, resulted in falsely elevated phosphate readings. Emphasized the need for proper flushing protocols.
C [11]	Case report of an 83‐year‐old woman on hemodialysis with a tunneled central venous catheter. Investigated high serum phosphate levels by analyzing blood samples and conducting in vitro experiments.	Contamination of blood samples with phosphate‐containing solutions, such as alteplase, used to lock catheters, led to falsely elevated serum phosphate levels. Emphasized proper flushing protocols.
D [3]	Case report of a patient on hemodialysis with a central venous catheter locked with alteplase. Investigated the cause of spurious hyperphosphatemia by analyzing blood samples.	Alteplase as a catheter locking solution can cause spurious hyperphosphatemia if blood samples are contaminated. Highlighted importance of flushing before sample collection.
E [12]	Case report of a patient with Waldenström's Macroglobulinemia. Investigated spurious hyperphosphatemia by analyzing blood samples.	Contamination of blood samples with heparin, which contains phosphate as a buffer, resulted in falsely elevated phosphate readings. Emphasized proper flushing protocols.
F [5]	Case reports of two pediatric patients on hemodialysis with central venous catheters. Investigated incidents of unexpectedly high serum phosphate levels and pre‐analytical errors.	Inadequate flushing of hemodialysis catheters led to blood sample contamination with phosphate‐containing anticoagulants, such as heparin and alteplase, causing falsely elevated phosphate levels. Highlighted adherence to proper blood collection protocols.
G [4]	Case report of a patient with chronic kidney disease who developed spurious hyperphosphatemia. Investigated potential causes, including sample contamination.	Contamination of blood samples with alteplase, used as an anticoagulant in hemodialysis, led to falsely elevated phosphate levels. Emphasized awareness of this potential interference.
Current Case	Case report of an end‐stage renal disease patient on hemodialysis via a central venous catheter who developed a falsely elevated phosphate level following alteplase instillation into the catheter.	Contamination of a blood sample with residual alteplase in a hemodialysis catheter led to falsely elevated phosphate levels. Emphasized the importance of adequate flushing and confirmatory testing to avoid misinterpretation.

To minimize such errors, the following preventive measures are recommended. This case supports the incorporation of stricter sampling protocols into clinical practice guidelines for dialysis units, particularly when using thrombolytic agents like alteplase:

Ensure proper catheter flushing before blood draws: After Cathflo administration, at least 10–20 mL of normal saline should be aspirated and discarded before obtaining a blood sample. This reduces the risk of alteplase contamination [13].

Confirm unexpected phosphate elevations with repeat testing: If an unexpectedly high phosphate level is obtained, a venous blood sample from a separate site (e.g., peripheral venipuncture) should be performed to rule out laboratory artifact [11].

Consider clinical context before modifying treatment: If a phosphate elevation is inconsistent with prior trends and lacks associated clinical signs, clinicians should suspect a pre‐analytical error rather than immediately altering the patient's dialysis prescription or phosphate binder regimen [14].

This case serves as a reminder for nephrologists, dialysis nurses, and laboratory personnel to be aware of catheter‐related pre‐analytical errors, particularly in hemodialysis patients who require precise phosphate management. Based on this case and prior reports, we hypothesize that the magnitude of phosphate elevation may be influenced by the concentration of alteplase used, the duration of dwell time, and the volume of residual solution remaining in the catheter at the time of sampling.

Spurious hyperphosphatemia from residual Cathflo in dialysis catheters is an underrecognized laboratory error in ESRD patients. As phosphate control is vital in dialysis care, false elevations may lead to unnecessary interventions. Proper catheter flushing, confirmatory sampling, and clinical correlation are essential to ensure accurate assessment and prevent mismanagement.

## Author Contributions

**Ahmad Matarneh:** conceptualization, writing – original draft, writing – review and editing. **Sundus Sardar:** writing – original draft, writing – review and editing. **Omar Salameh:** writing – original draft, writing – review and editing. **Muhammad Abdulbasit:** writing – original draft, writing – review and editing. **Wardah Iqbal:** writing – original draft, writing – review and editing. **Monika Koirala:** writing – original draft, writing – review and editing. **Taryn Millete:** writing – original draft. **Ronald Miller:** writing – original draft, writing – review and editing. **Nasrollah Ghahramani:** writing – original draft, writing – review and editing. **Navin Verma:** writing – original draft, writing – review and editing.

## Ethics Statement

Penn State ethical committee does not require ethical approval for reporting individual cases.

## Consent

Written informed consent was obtained from the patient for his anonymized information to be published in this article.

## Conflicts of Interest

The authors declare no conflicts of interest.

## Data Availability

The data that support the findings of this study are available from the corresponding author upon reasonable request.
