# Correction to ‘Spurious Hyperphosphatemia From Alteplase‐Treated Catheter: A Case Report and Literature Review’

**DOI:** 10.1002/ccr3.71936

**Published:** 2026-01-26

A. Matarneh, S. Sardar, O. Salameh, M. Abdulbasit, W. Iqbal, M. Koirala, T. Millete, R. Miller, N. Ghahramani, N. Verma, “Spurious Hyperphosphatemia From Alteplase‐Treated Catheter: A Case Report and Literature Review,” *Clinical Case Reports* 13, no. 8 (2025): e70665, https://doi.org/10.1002/ccr3.70665.

In the published version of this article, errors were identified in the reference list, limited to bibliographic citation details, including incorrect or incomplete author names, journal titles, publication years, volume or issue numbers, and page ranges. During the correction process, the entire reference list was reviewed against the original source publications and corrected where necessary to ensure accuracy and consistency with the cited literature. No references were added or removed, and no changes were made to the in‐text citations, scientific content, data interpretation, or conclusions of the article. The authors apologize for these errors.

Reference #9

In Published paper

A. M. Ball, W. D. Tobler, L. D. Ferrell, and L. Chan, “Spurious Hyperphosphatemia due to Sample Contamination With Heparin From Arterial Catheters,” *American Journal of Kidney Diseases* **44**, no. 2 (2004): 367–371, https://doi.org/10.1053/j.ajkd.2004.04.033.

In new version

Schiller B, Virk B, Blair M, Wong A, Moran J. Spurious Hyperphosphatemia in Patients on Hemodialysis With Catheters. *American Journal of Kidney Diseases*. 2008 Sep 1;52(3):617–20.

Reference #14

In Published paper

T. Sav, H. Yilmaz, A. V. Kara, E. Memisoglu, and O. Cakmak Oksuz, “Spurious Hyperphosphatemia in Hemodialysis Patients: Effect of Catheter‐Locking Solutions,” *Hemodialysis International* **19**, no. 2 (2015): 317–320, https://doi.org/10.1111/hdi.12250.

In new version

Sombolos K, Bamichas G, Hatsiou V, Fragidis S, Rizos A, Natse T, Fytili C. Alteplase as Hemodialysis Catheter Locking Solution and Spurious Hyperphosphatemia. *The Journal of Vascular Access*. 2011 Jul;12 (3):269.

